# Management of complications associated with percutaneous left atrial appendage closure with or without ablation: experience from 512 cases over a 4-year period

**DOI:** 10.3389/fcvm.2024.1388024

**Published:** 2024-07-23

**Authors:** Qi Zou, Cheng Jiang, Pengyang Lin, Yangyang Yu, Jiazheng Li, Feng Zhao, Hao Hu, Shougang Sun

**Affiliations:** Department of Cardiology, Lanzhou University Second Hospital, Lanzhou, China

**Keywords:** left atrial appendage closure, procedural-related complications, device-related thrombus, management strategy, atrial fibrillation

## Abstract

**Background:**

Percutaneous left atrial appendage closure (LAAC) serves as an alternative prophylactic strategy for patients with non-valvular atrial fibrillation (AF) who cannot undergo anti-coagulation therapy. Proper management of associated complications is crucial to enhancing the procedure's success rate and mitigating perioperative risks and adverse events during follow-up.

**Aims:**

This study aims to summarize our center's experience and strategies in managing procedural-related complications encountered in 512 cases of LAAC with or without ablation for AF conducted from January 2020 to December 2023.

**Results:**

We identified 11 significant intervention-requiring complications associated with LAAC with or without Ablation procedure. These included three cases of intraoperative thrombosis, three instances of pericardial effusion or tamponade, one case of device-related thrombosis, one peri-device leak, one systemic embolism, one bleeding episode, and one additional device-related complication. The categorization of intraoperative thrombosis was as follows: one patient exhibited heparin resistance; one experienced thrombosis due to prolonged device implantation during the LAAC with ablation procedure; and one had unexplained intraoperative thrombosis. The pericardial effusion or tamponade likely resulted from damage to the atrial appendage during LAAC device insertion. Two patients encountered device-related thrombosis and systemic embolism events possibly caused by non-standard postoperative antithrombotic medication use; one patient's peri-device leak may have resulted from incomplete endothelialization of the occluder post-surgery; one patient experienced postoperative bladder bleeding; and one patient's device-related complications occurred due to a dislodged strut frame that damaged the left atrial appendage, leading to pericardial effusion. Our proactive interventions enabled all patients with these surgical-related complications to be safely discharged, with subsequent follow-ups showing no adverse events.

**Conclusion:**

Implementing targeted interventions for immediate procedural-related complications during the LAAC with or without ablation procedures enhances procedural success rates, diminishes postoperative mortality and patient disability, and bolsters stroke prevention efforts. This approach underscores the importance of a strategic response to complications, affirming the procedure's viability and safety in managing non-valvular AF in patients contraindicated for anticoagulation.

## 1 Introduction

Atrial fibrillation (AF) stands as one of the most prevalent cardiac arrhythmias today, witnessing a global surge. Data from the Framingham Heart Study reveal a threefold increase in its prevalence over the past five decades (1). Projections indicate that by 2050, AF will afflict at least 72 million individuals across Asia. To prevent stroke events in AF patients, long-term use of anticoagulants like warfarin and DOACs is often required. However, these medications can adversely affect quality of life, causing gastrointestinal issues, allergies, and potential liver and kidney damage (2). The therapeutic window for these drugs is narrow, demanding meticulous dosage control, insufficient amounts render the therapy ineffective, whereas excessive dosages predispose patients to hemorrhagic complications (3). Moreover, certain patient cohorts either exhibit contraindications to enduring anti-coagulation therapy or experience thromboembolic episodes despite rigorous adherence to it. The innovation of percutaneous left atrial appendage closure (LAAC) has emerged as a promising resolution to these quandaries (4).

The left atrial appendage (LAA) is the main site for thrombus formation in AF patients. The LAAC procedure reduces this risk by blocking blood flow to the LAA with a closure device. The 2012 European Society of Cardiology (ESC) guidelines support LAAC as an alternative for patients at high stroke risk who cannot tolerate long-term oral anticoagulation (Class IIb, Level B) (5). This recommendation gained further impetus in 2015 following the U.S. Food and Drug Administration's (FDA) approval of the WATCHMAN device, subsequent to two critical randomized controlled trials, thereby catalyzing the global adoption of this technique (6). An innovative approach has emerged in the form of a LAAC with ablation procedure, offering a comprehensive therapeutic strategy. This dual intervention may confer superior benefits over standalone procedures, particularly for symptomatic AF patients with both elevated ischemic stroke risk and indications for ablation (7). Presently, an assortment of LAAC devices, chiefly categorized as internal plug types (e.g., Watchman/Watchman FLX) and external cover types [e.g., LAmbre and LACbes in China, AMPLATZERTM Cardiac Plug (ACP)/Amulet in the U.S.], are employed globally. Advances in medical technology and accumulative clinical experience have established LAAC as a secure and potent preventive measure. Large-scale clinical trials underscore the procedure's efficacy in preventing strokes (8). Nonetheless, continuous investigations into LAAC have uncovered several procedural complications, including pericardial effusion/tamponade, air embolism, thromboembolism, device dislodgement, and device-related thrombosis (DRT). These findings necessitate a heightened focus on effective complication management (9).

Our study encapsulates insights derived from managing perioperative complications in 11 distinct LAAC procedures within a single institution, underscoring the criticality of prompt and adept complication management. Such interventions enhance procedural success rates, optimize patient prognoses, and curtail potential adverse outcomes.

## 2 Methods

### 2.1 Patient population

This study encompasses cases from the Departments of Cardiology at Lanzhou University Second Hospital, China, a tertiary institution incorporating medical services, education, research, a specialized interventional catheterization unit, and a Coronary Care Unit (CCU). Annually, the center conducts approximately 130 LAAC procedures. In adherence to the local ethics committee's stipulations, all participants provided written informed consent. Patient follow-ups, conducted from January 2020 to December 2023, comprised in-person consultations, hospitalization records, and survey data. During this period, we completed a total of 512 cases of LAAC/LAAC with ablation procedure. According to the follow-up data of patients, the occurrence of complications at our center was as follows: Intra-operative Device Thrombosis Events: 3/512 (0.59%), Pericardial Effusion/Tamponade Events: 4/512 (0.78%), Device-Related Thrombosis Events: 7/512 (1.37%), Peri-Device Leak Events: 5/512 (0.98%), Systemic Embolism Events: 1/512 (0.20%), Device-related Complications Events: 1/512 (0.20%), Bleeding Events: 3/512 (0.59%). The specific annual LAAC volume and complication rates at our centre are shown in Table 1. No other complications were observed in our patient cohort at our center. In this study, 11 representative patients with complications were further identified for reporting and discussion of complication management strategies. Patient baseline information and summaries are specifically shown in Table 2. This report emphasizes severe complications necessitating management during either the perioperative period or subsequent follow-ups. A clinical events committee, consisting of two cardiologists and an adjudicating third expert, independently assessed complication events associated with the perioperative phase and follow-up sessions.

**Table 1:** Number of LAAC and complication rates at our centre 2020–2023.

	2020	2021	2022	2023
Number of LAAC	137	121	100	154
Intra-operative device thrombosis (%)	2 (1.5%)	1 (0.8%)	0 (0.0%)	0 (0.0%)
Device-related thrombosis (%)	3 (2.2%)	1 (0.8%)	2 (2.0%)	1 (0.6%)
Pericardial effusion/tamponade (%)	2 (1.5%)	0 (0.0%)	2 (2.0%)	0 (0.0%)
Peri-device leak (%)	1 (0.7%)	2 (1.7%)	1 (1.0%)	1 (0.6%)
Systemic embolism (%)	0 (0.0%)	1 (0.8%)	0 (0.0%)	0 (0.0%)
Bleeding (%)	0 (0.0%)	2 (1.7%)	0 (0.0%)	1 (0.6%)
Device-related complications (%)	0 (0.0%)	0 (0.0%)	1 (1.0%)	0 (0.0%)

**Table 2:** Summary of patient baseline and follow-up information.

	Age	Gender	Weight	Hypertension	Diabetes	Stroke	Hemorrhage	Pre-operative antithrombotic strategy	CHA_2_DS_2_–VASc score	HAS-BLED score	LAA diameter (mm)	LAA depth (mm)	LAA morphology	Device (size mm)	Post-operative antithrombotic strategy	Follow-up	Timing of complication	Complication
1	59	Male	50	No	No	No	No	Non-thrombotic	2	2	21	20	Chicken-wing	LACbes (24–30)	Rivaroxaban (20 mg daily), aspirin (100 mg daily)	TEE	Perioperative	Intra-operative device thrombosis
2	59	Male	72	Yes	Yes	Yes	No	Rivaroxaban (20 mg daily)	5	3	18	22	Windsock	WATCHMAN (22)	Rivaroxaban (20 mg daily), aspirin (100 mg daily)	TEE	Perioperative	Intra-operative device thrombosis
3	45	Female	55	No	No	Yes	No	Non-thrombotic	4	2	33	24	Chicken-wing	LACbes (28–34)	Rivaroxaban (20 mg daily), aspirin (100 mg daily)	TEE	Perioperative	Intra-operative device thrombosis
4	72	Female	70	Yes	No	Yes	No	Aspirin (100 mg daily)	6	5	22.8	27.9	Chicken-wing	WATCHMAN (32)	Rivaroxaban (20 mg daily), aspirin (100 mg daily)	TEE	38 days post-operative	DRT
5	68	Female	53	No	No	Yes	No	Non-thrombotic	5	2	20	10	Reversed chicken-wing	Atrial septal occluder (10)	Rivaroxaban (20 mg daily), aspirin (100 mg daily)	TEE	Perioperative	Pericardial effusion/tamponade
6	75	Female	60	No	No	Yes	No	Non-thrombotic	5	2	10	21	Cactus	LACbes (18–24)	Rivaroxaban (20 mg daily), clopidogrel (75 mg daily)	TEE	Perioperative	Pericardial effusion/tamponade
7	69	Female	69	No	No	No	No	Non-thrombotic	3	2	17	21	Chicken-wing	WATCHMAN (21)	Rivaroxaban (20 mg daily), clopidogrel (75 mg daily)	TEE	Perioperative	Pericardial effusion/tamponade
8	64	Male	60	No	No	No	No	Non-thrombotic	2	2	17	21	Chicken-wing	WATCHMAN (21)	Rivaroxaban (20 mg daily), aspirin (100 mg daily)	CCTA	50 days post-operative	PDL
9	76	Male	55	Yes	No	Yes	No	Rivaroxaban (20 mg daily)	6	6	14	16	Windsock	WATCHMAN (21)	Clopidogrel (75 mg daily), aspirin (100 mg daily)	TTE	180 days post-operative	Systemic embolism
10	64	Male	50	Yes	No	No	No	Non-thrombotic	4	2	19	21	Chicken-wing	WATCHMAN (23)	Rivaroxaban (15 mg daily), aspirin (100 mg daily)	TEE	11 days post-operative	Bleeding
11	75	Female	55	Yes	No	No	No	Non-thrombotic	2	3	27	26	Chicken-wing	WATCHMAN (33)	Rivaroxaban (15 mg daily), aspirin (100 mg daily)	CCTA	30 days post-operativ	Device-related complications

### 2.2 LAAC procedure and LAAC with ablation procedure

The criteria for LAAC, or the LAAC with ablation procedures comply with prevailing guidelines and recommendations. Under general anesthesia, LAAC procedures are performed on patients with AF, following comprehensive preoperative evaluations that include imaging studies, blood assays for cardiac and LAA morphology, cardiac functionality, coagulation status, hepatic and renal functions, complete blood counts, and other relevant clinical indicators. Adhering to international surgical guidelines and manufacturers' protocols for LAAC devices, the selection of specific LAAC procedures and devices resides with the attending specialist. Devices deployed include Watchman™ (Boston Scientific, MA, USA) or LACbes device (PushMed, SHH, CHN). The LAAC with ablation strategy involves a concurrent LAAC procedure and radiofrequency catheter ablation (RFCA). In cases where suitable candidates consent to the approach, pulmonary vein isolation is additionally undertaken. During RFCA, parameters such as the modality of ablation energy, the employment of cardiac navigational systems, ancillary catheters for electrophysiological mapping, and the sequence of procedures are operator-determined. The ablation endpoint is validated by the obliteration of pulmonary vein potentials and the confirmation of bidirectional blockage within the ablation circles or lines. Post-procedural management and antithrombotic therapies are individualized, aligning with each patient's unique risk profile, under the presiding specialist's guidance.

### 2.3 Definition of perioperative complications and subsequent events during follow-up

Our criteria for identifying perioperative complications and related events during follow-up adhere to the standards set forth in the Munich Consensus Document. This comprehensive guideline was collaboratively established by experts from Europe, North America, the European Society of Cardiology, the medical device industry, and various clinical specialists. It provides detailed definitions for parameters and endpoint events crucial for evaluating LAAC clinical studies (10). The specific definitions of the relevant events are as follows.

#### 2.3.1 Pericardial effusion/tamponade

Pericardial effusion, which may or may not progress to tamponade, stands as a significant potential complication following endoluminal catheter procedures. Clinically significant pericardial effusions are characterized by any of the following criteria: (1) Necessitation of therapeutic pericardiocentesis; (2) Induction of surgical intervention; (3) Requirement for blood transfusion, (4) Precipitation of shock and/or fatality. The temporal parameters defining the onset of pericardial effusion are categorized as follows: (1) Intraprocedural—arising during the index procedure. (2) Acute—manifesting within 48 h post-procedure. (3) Late—developing beyond 48 h post-procedure.

#### 2.3.2 Device-related complications

Device-related complications include a range of issues, including: device-related thrombosis (DRT), device embolism, erosion, adverse interactions with adjacent structures (e.g., circumflex coronary artery, mitral valve, pulmonary artery, pulmonary vein), structural fracture, puncture or laceration, infection, and endocarditis.

#### 2.3.3 Systemic embolism

Acute vascular insufficiency or occlusion of the extremities or any non-CNS organ is identified by clinical, imaging, or surgical/autopsy evidence of arterial occlusion in the absence of other likely mechanisms (e.g., trauma, atherosclerosis, or instrumentation). When there is a presence of prior peripheral artery disease, angiographic, surgical, or autopsy evidence is required to show abrupt arterial occlusion.

#### 2.3.4 Stroke and transient ischemic attack (TIA)

A stroke is identified as an acute event marked by focal or global neurological impairment attributable to hemorrhagic or ischemic vascular disruptions impacting the brain, spinal cord, or retina. In contrast, a transient ischemic attack (TIA) is distinguished from an ischemic stroke by the temporary nature of neurological symptoms, which resolve within 24 h, and the absence of acute cerebral infarction on subsequent imaging evaluations.

### 2.4 Perioperative and postoperative imaging follow-up

#### 2.4.1 Transesophageal echocardiography (TEE)

TEE is systematically conducted within 48 h preceding the LAAC to meticulously assess several parameters: the LAA's anatomical characteristics (including morphology, orifice dimensions, depth, lobe distribution, and muscular architecture), thrombus presence or spontaneous echocardiographic contrast, interatrial septal features, and LAA emptying velocity alongside systolic function. TEE provides detailed imagery of the septum's superior, inferior, anterior, and posterior aspects. The interatrial septum puncture, guided by TEE, typically utilizes a right anterior oblique 30-degree orientation and a caudal angle of 20 degrees or other suitable angles. The selection of the appropriate LAAC device is informed by both digital subtraction angiography (DSA) and TEE, gauging the LAA orifice's width and viable depth. Upon satisfying the release criteria, the device is fully deployed. Subsequent to this, multi-angle TEE re-evaluation is essential to confirm the device's positioning, its impact on neighboring structures (such as pulmonary veins and mitral valves), and to identify any pericardial effusion. Both the compression ratio and any residual flow are meticulously documented via TEE.

#### 2.4.2 Transthoracic echocardiography (TTE)

TTE, performed typically a week before LAAC, serves as a standard examination to delineate left ventricular systolic function, left atrial dimensions, atrial septal characteristics, cardiac valve conditions, pulmonary arterial pressure, and any pericardial effusion. During subsequent postoperative evaluations, TTE is instrumental not only in verifying the LAAC device's stability and pinpointing pericardial effusion but also in scrutinizing both systolic and diastolic cardiac functions, evaluating valve integrity, and detecting any anatomical alterations.

#### 2.4.3 Cardiac computed tomography angiography (CCTA)

For patients unable to undergo TEE due to intolerance or esophageal pathology, CCTA serves as an alternative for both preoperative assessments and postoperative follow-ups in the LAAC context. It is pivotal to recognize that LAA orifice dimensions obtained via CCTA generally exceed those acquired through TEE by approximately 3 mm. Moreover, the sensitivity and specificity of CCTA in identifying thrombi within the left atrium and LAA are inferior to those of TEE. Consequently, CCTA findings necessitate a judicious and cautious interpretation.

### 2.5 Perioperative management

Standard preoperative protocols for cardiovascular interventions are meticulously observed, including the removal of dentures, establishment of venous access, and an 8-h fast from food, liquids, and oral medications prior to the procedure. A thorough review of each patient's clinical profile and pertinent test results is essential to reaffirm the indications, contraindications, or specific criteria excluding LAAC. Additionally, understanding the anatomical nuances of the LAA, such as orifice diameter, feasible depth, muscular architecture, and lobing, is crucial, along with assessing the dimensions of both atria, the presence and severity of any pericardial effusion, and historical medical interventions like atrial septal repairs or occlusions. Any concurrent conditions, such as pulmonary surgeries, thoracic anomalies, or cardiac transpositions, must be noted to anticipate the complexity of atrial septal punctures and to guide the selection of suitable LAA devices, thereby ensuring a comprehensive surgical strategy with viable contingency plans anti-coagulation commences upon the patient's eligibility confirmation for LAAC and their consent to proceed. For those on DOACs, administration continues until the day before the procedure and is withheld on the morning of the intervention. Those on warfarin should have their international normalized ratio (INR) monitored daily, continuing intake until one day before and suspending it on the procedure's morning. Patients not on pre-procedural anti-coagulation are provided low-molecular-weight heparin subcutaneously upon admission, which is discontinued in the morning before the procedure.

### 2.6 Follow up

The schedule for clinical follow-ups is determined by the attending physician's judgment. Typically, patients are advised to undergo follow-up TEE or TTE at 45 days, 3 months, and 6 months postoperatively. For patients unable to tolerate TEE due to esophageal conditions or probe insertion difficulties, CCTA is a viable alternative at both 3 and 6 months post-operation. These assessments are crucial for evaluating the status and severity of pericardial effusion, verifying the LAAC device's positioning, inspecting the adjacent anatomical structures of the LAA, identifying any residual leaks after LAAC, detecting DRT, and confirming endothelialization.

## 3 Results

### 3.1 Management of intra-operative device thrombosis events

**Figure 1:**
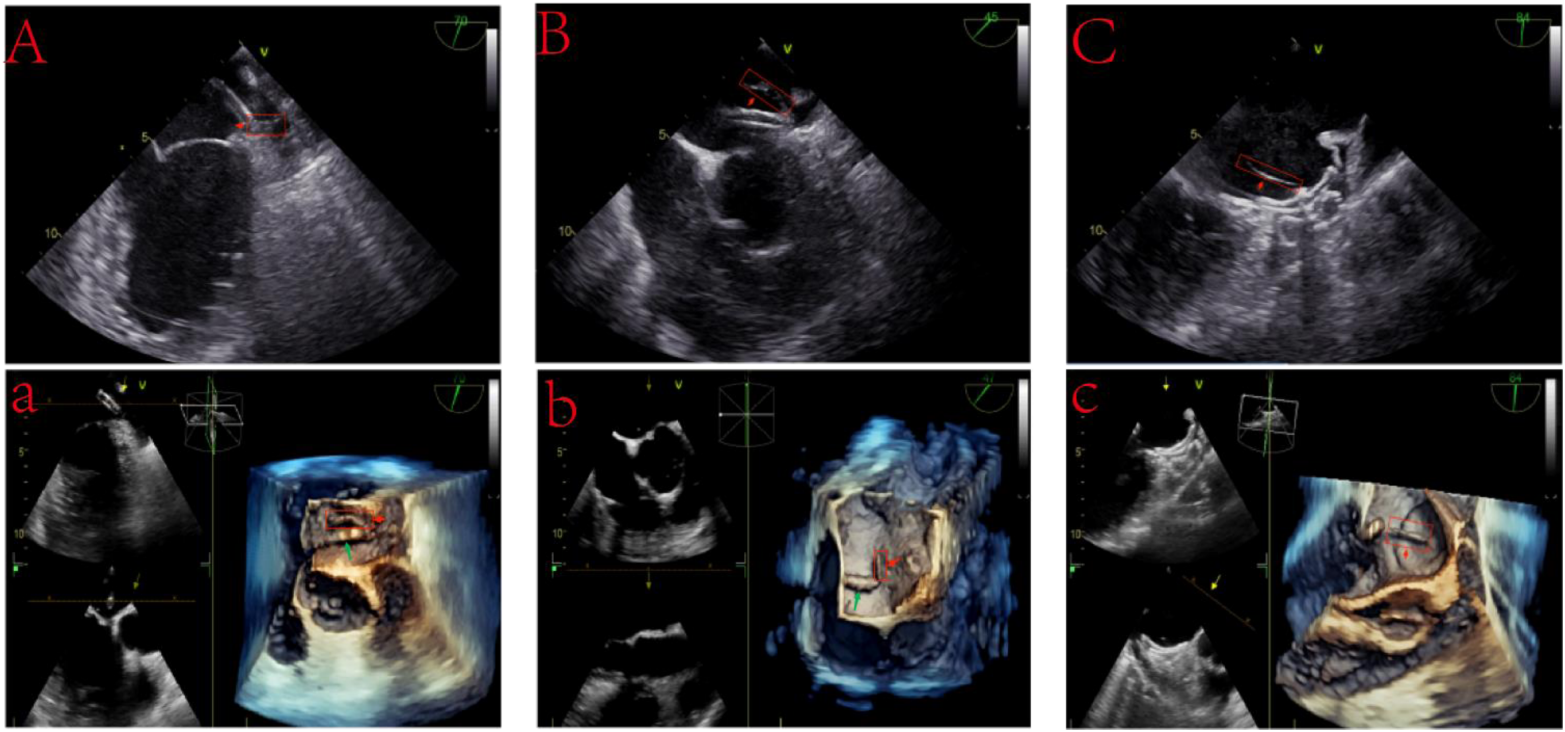
Intraoperative thrombosis during left atrial appendage closure. (**A**) A 2D TEE image reveals an accumulating flocculent substance at the catheter's tip, identified as a thrombus (15 mm × 5 mm). (**A**) 3D TEE visualization highlights the thrombus formation (red arrows) at the catheter's head, with the catheter itself marked by green arrows. (**B**) Post-deployment of a WATCHMAN device, a thrombus (20 mm × 5 mm) is noted at the catheter's tip during its extraction phase. (**B**) 3D TEE imagery captures the thrombus (red arrows) and the catheter (green arrows). (**C**) Following the LACbes device deployment, a distinct linear thrombus (25 mm × 2 mm) is observed along the device's central rivet. (**C**) Enhanced 3D TEE imaging showcases the thrombus detailed in (**C**), with red arrows pointing to its formation.

Three patients, initially thrombus-free in the LAA as per preoperative TEE, developed thrombi during the procedure. One case involved a 59-year-old male with AF and no anti-coagulation history before admission. Due to the high cost of medication, which led to poor adherence, we performed LAAC on this patient to prevent embolic events. Following a comprehensive preoperative assessment and deliberation, we proceeded with LAAC. Post-general anesthesia, we accessed the right femoral vein and initiated heparinization with 3,000 units. A TEE-guided transseptal puncture was successfully executed, followed by an additional 3,500 units of heparin. And then we put in a 14F catheter that carries the left atrial appendage occlusion device. In preparation for further operations, TEE detected flocculent material on the catheter's distal end, indicative of thrombus formation (Figure 1A). Immediate ACT monitoring showed 160 s, prompting an additional 2,000 units of heparin. Approximately 5 min later, ACT read 180 s. Suspecting heparin resistance, we administered another 2,000 units of heparin and aspirated the thrombus via catheter. TEE confirmed the subsequent dissolution of flocculation, and ACT increased to 260 s. To mitigate intraoperative cerebral embolism, we employed a cerebral protection system (CPS, Covidien, MA, USA), placed through femoral access, deploying proximal filters in both internal carotid arteries. A 24–30 mm LACbes device (PushMed, SHH, CHN) was then positioned within the LAA. After recording an ACT of 234 s, we retracted the CPS and removed the catheter from the right femoral vein. Post-procedure analysis of the CPS filter revealed no thrombotic material or tissue debris. The patient exhibited no postoperative systemic embolism or ischemic stroke symptoms. He was discharged on the fifth postoperative day and prescribed a 12-week regimen of rivaroxaban (20 mg daily) and aspirin (100 mg daily) for post-discharge anti-coagulation and antiplatelet therapy. No complications detected on follow-up.

In the second patient diagnosed with AF, despite being on DOAC anticoagulation therapy, the patient experienced a stroke event, indicating DOAC failure. Based on current research, for patients with non-valvular AF, the occurrence of ischemic stroke events despite prescribed DOAC therapy is termed “DOAC failure” (11). Given this, our patient experienced a DOAC failure event. To prevent further stroke events, we performed the LAAC with ablation procedure. Post-assessment of the LAA's dimensions and morphology, we positioned a 22-mm WATCHMAN device (Boston Scientific, MA, USA) within the LAA, achieving successful deployment. However, during preparatory steps for extraction, TEE identified thrombus formation on the catheter's distal end, potentially attributed to the prolonged stay of the catheter in the vessel during the LAAC with ablation procedure (Figure 1B). Following CPS deployment, we administered a 50 mg prourokinase (Shanghai Tasly Pharmaceutical Co, Ltd.) thrombolytic treatment, with subsequent ACT recorded at 260 s. TEE post-thrombolysis confirmed thrombus resolution at the catheter's distal end. Post-procedural CPS filter analysis revealed no thrombotic or tissue debris. After the discharge, the patient embarked on a 12-week anti-coagulation and antiplatelet regimen, comprising rivaroxaban (20 mg daily) and aspirin (100 mg daily), with no complications detected on follow-up.

In the third AF patient, due to the patient's self-perceived symptom improvement, they stopped the medication on their own. Additionally, insufficient understanding of DOACs anticoagulation led to poor medication adherence. we introduced a 28–34 mm LACbes device into the LAA following transseptal puncture and an initial 3,000-unit heparin dose. The device was successfully deployed. However, prior to catheter removal, TEE detected a linear thrombus at the device's rivets (Figure 1C). Immediate ACT was 196 s, prompting an additional 5,000-unit heparin dose. ACT readings taken at 5-min intervals post-supplementation were 224 s and 256 s, following 3,000 additional units of heparin. After positioning CPS in the bilateral internal carotid arteries, we administered 50 mg of prourokinase, resulting in thrombus dislodgment within 2 h. CPS retrieval and filter analysis identified a fragmented white thrombus in the right filter. Subsequently, the patient exhibited an unsteady gait, headaches, and nausea, prompting suspicions of an intraoperative thrombus obstructing the cerebellar-supplying branch vessels via the vertebral artery. However, a brain MRI did not detect any occlusion of the PICA or AICA. Instead, it revealed an abnormal signal area in the right parietal lobe, with high signal on DWI sequences and low signal on ADC sequences, suggesting a transient ischemic event due to incomplete capture of small thrombi by the CPS device. Following stabilization, the patient was discharged, commencing standard postoperative anti-coagulation and antiplatelet therapy, including rivaroxaban (20 mg daily) and aspirin (100 mg daily). No complications detected on follow-up.

### 3.2 Management of device-related thrombosis events

**Figure 2:**
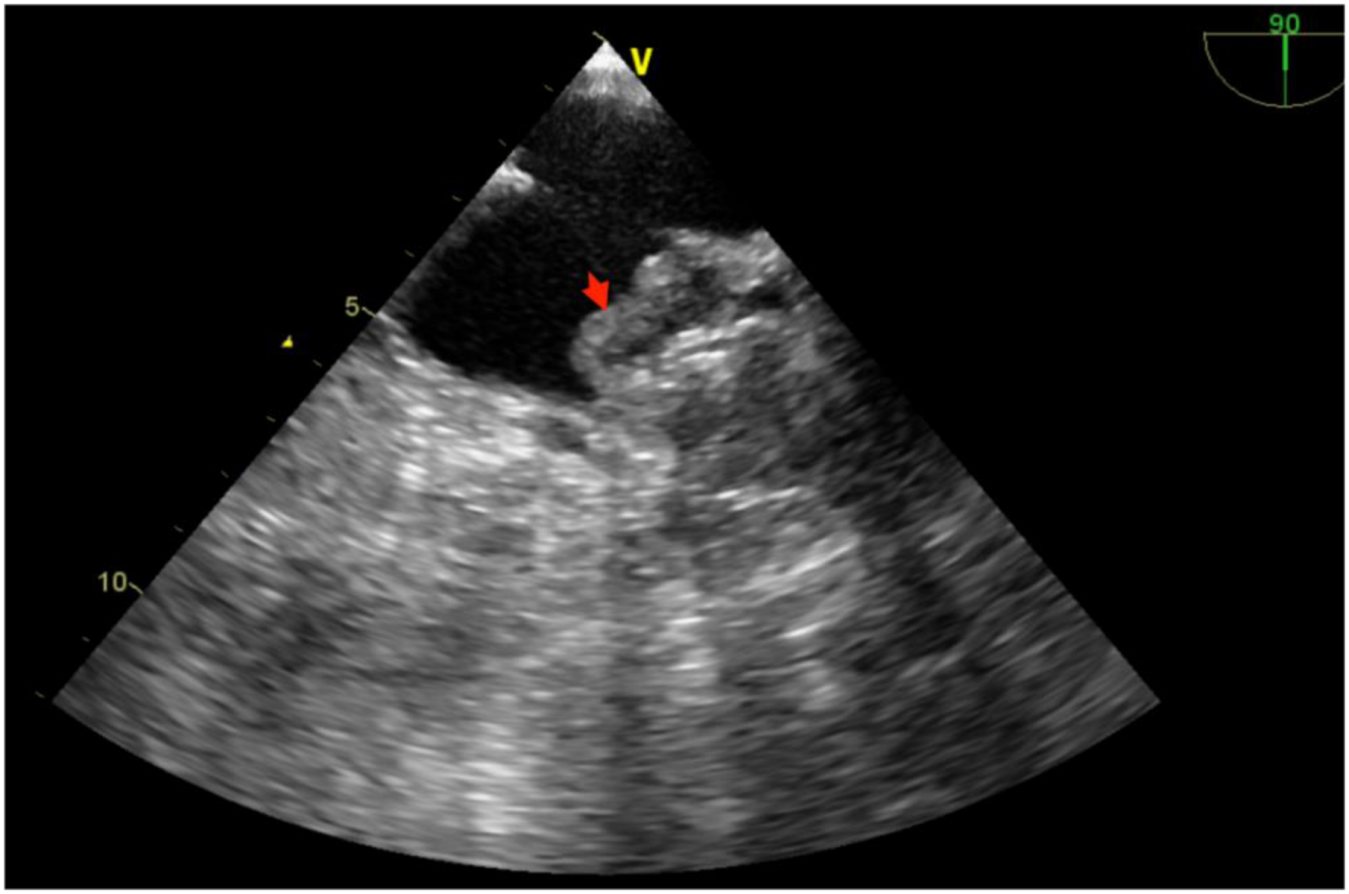
DRT events during follow-up. Red arrows indicate thrombus on the surface of the occluder.

A 72-year-old with a 4-year history of AF and prior long-term aspirin use, with a HAS-BLED score of 5 (Hypertension, Abnormal liver function, Stroke, Elderly, long-term aspirin use), requested LAAC due to a fear of bleeding complications from anticoagulants. The patient underwent a successful LAAC using a 32-mm WATCHMAN device, installed accurately without any residual leaks. Postoperatively, the patient commenced a 12-week regimen of rivaroxaban (20 mg daily) and aspirin (100 mg daily) for combined anticoagulation and antiplatelet therapy. On day 38 post-LAAC, a TEE examination identified a medium-low density echo cluster on the surface of the left atrium and occluder, indicative of potential DRT (Figure 2). Further investigation revealed that, similar to a prior case, this patient had independently ceased taking aspirin postoperatively, continuing only with rivaroxaban. The antithrombotic treatment was promptly reinstated, and after consistent medication for 94 days, a subsequent TEE confirmed the resolution of DRT, showing no abnormal echoes outside the occluder in the left atrium and LAA. Throughout the follow-up period, no ischemic strokes, TIA, or hemorrhagic complications were reported.

### 3.3 Management of pericardial effusion/tamponade events

**Figure 3:**
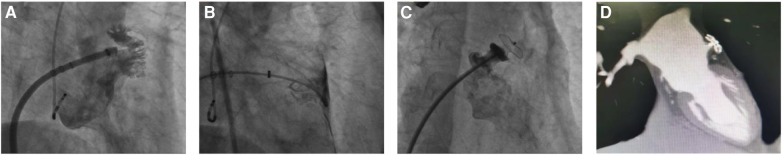
The LAAC procedure utilizing an atrial septal defect occluder on a “chicken-wing” shaped LAA. (**A**) DSA illustrates a superficial “chicken-wing” configuration of the left auricle. (**B**) Post-puncture of the LAA, instead of immediate catheter retraction following contrast medium diffusion, the catheter was advanced further into the pericardial space through the breach. (**C**) Utilizing this strategically retained catheter, an atrial septal defect occluder was deployed atop the LAA opening. Subsequent DSA confirmed the halt of bleeding and effective LAA closure by the occluder. The red arrow highlights the 10-mm atrial septal defect occluder positioned within the LAA. (**D**) On review at 1 month postoperatively, CCTA suggested good position of the occluder, and no adverse events such as residual leakage were detected.

Among the patients presenting with pericardial effusion or tamponade during LAAC at our facility, four instances were notable, potentially attributable to atrial appendage injury during device placement. The initial case concerned a 68-year-old female with AF, previously diagnosed with tuberculosis, cerebral embolism, and TIA. The patient had a CHA_2_DS_2_-VASc score of 5 (Prior Stroke, peripheral artery disease, age 68, female). This patient had previously experienced a stroke and remained at high risk for subsequent stroke events. Additionally, the patient had cognitive impairment leading to poor medication adherence. Upon admission, a preoperative TEE ruled out a left atrial thrombus, and LAAC was deemed suitable after evaluation. The patient's “pendulous heart” morphology complicated septal puncture, as the elevated atrial septum position necessitated precise puncture site selection. Despite utilizing the right internal jugular vein for access and a CS10 pole electrode in the coronary sinus for guidance, the puncture remained high post-procedure. LAA DSA identified a “reversed chicken wing” morphology, characterized by a diminutive ostium and limited LAA depth (Figure 3A). Intending to optimize left atrial appendage occlusion device placement, we navigated the pigtail catheter farther into the LAA. However, DSA found the catheter had breached the LAA, penetrating the pericardial space (Figure 3B), coinciding with a blood pressure decline to 70/40 mmHg. The patient suddenly developed a pericardial tamponade. Emergency subxiphoid pericardial drainage was initiated, stabilizing the patient's hemodynamics. To expedite stiffened guide-wire insertion into the pericardium, we maintained the pigtail catheter's position, deploying a 10-mm atrial septal occluder (Lifetech Scientific, SZ, CHN) from the pericardial space to the LAA, followed by catheter retraction and occluder release (Figure 3C). Subsequent assessments indicated successful occlusion, with no pericardial blood ingress or effusion recurrence. After the procedure, the patient embarked on a 12-week dual-therapy regimen consisting of rivaroxaban (20 mg, twice daily) and aspirin (100 mg daily). A 33-day postoperative CCTA revealed no anomalies (Figure 3D). Further, a 135-day postoperative TEE detected no device-associated thrombosis or pericardial abnormalities, and a 208-day follow-up CCTA reevaluation showed no abnormality.

**Figure 4:**
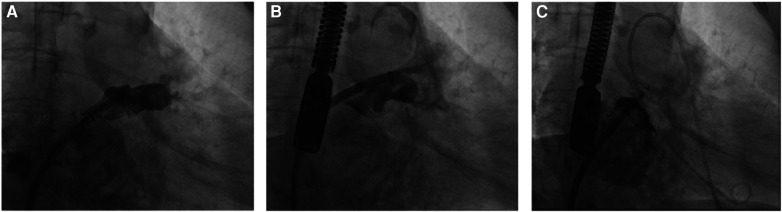
The cessation of bleeding following a temporary deployment of the LACbes device after inadvertent scratching of the LAA during the LAAC procedure. (**A**) The LAA undergoes angiographic assessment post-septal puncture, guiding the selection of a suitable occluder. (**B**) Intraoperative disturbance of the LAA surface resulted in discernible contrast spread into the pericardial space, indicating bleeding. (**C**) Following the provisional placement of the LACbes device, subsequent angiography revealed the absence of contrast seepage, confirming the halting of hemorrhage.

The second case involved a 75-year-old female evaluated for and subsequently undergoing the LAAC procedure. The patient had no prior history of taking anticoagulant medication and had a high risk of stroke (CHA2DS2-VASc score: 5, including Prior Stroke, age 75, female). This patient had previously experienced a stroke and remained at high risk for subsequent stroke events. Additionally, the prior stroke led to cognitive impairment, resulting in poor medication adherence. Therefore, we decided to perform LAAC to prevent further thromboembolic events. An 18–24 mm LACbes device was positioned within the LAA. During the device's deployment, the patient experienced a blood pressure decrease to 85/45 mmHg, concurrent with the emergence of a pericardial effusion identified on TEE. Our immediate response was a provisional device release to seal the perforation, simultaneously summoning cardiac surgical support to address the hemorrhage. Following the device's stabilization, we observed no further escalation in pericardial fluid accumulation (Figure 4), leading us to surmise that the bleeding had ceased due to the device implantation effectively sealing the puncture. The position of the occlusion device was assessed as good using TEE and the patient was discharged after his vital signs were stabilized. Patients are discharged from the hospital and commenced a 12-week regimen of rivaroxaban (20 mg daily) and clopidogrel (75 mg daily) for sustained anti-coagulation and antiplatelet therapy. No complications detected on follow-up.

**Figure 5:**
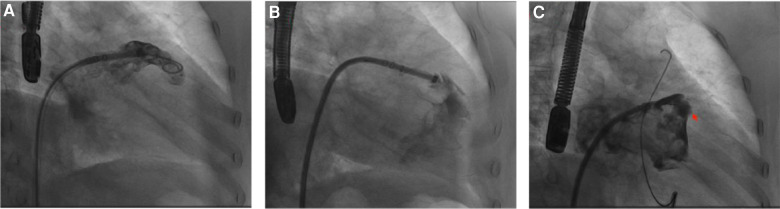
The effective application of a WATCHMAN device to seal a bleeding LAA breach. (**A**) Initial angiographic scrutiny is performed on the LAA following septal puncture, aiding in the appropriate choice of occluder. (**B**) A tear within the LAA is visualized with angiographic contrast dispersing into the pericardium, signaling internal bleeding. (**C**) Post-deployment of a WATCHMAN device to seal the LAA rupture, no further contrast leakage is observed within the pericardial area, signifying successful occlusion. The red arrow points to the WATCHMAN device, strategically positioned as a temporary measure.

The third instance concerned a 69-year-old female who found the cost of DOACs to be prohibitively expensive and was unwilling to use them long-term. Consequently, she requested the LAAC procedure. Following preoperative assessment, she received a 21-mm WATCHMAN device implanted within the LAA after septal puncture. The initial device deployment presented sub-optimal positioning, prompting a retrieval attempt for repositioning. During this process, the catheter inadvertently perforated the LAA. Echoing the previous patient's scenario, the hemorrhage was arrested when the device was temporarily released, sealing the LAA perforation and precluding further bleeding exacerbations. Subsequent TEE assessments verified the device's satisfactory placement (Figure 5). This intervention circumvented the necessity for more invasive open-heart surgery. Both patients procedural recovery, commenced a 12-week regimen of rivaroxaban (20 mg daily) and clopidogrel (75 mg daily) for sustained anti-coagulation and antiplatelet therapy. No complications detected on follow-up.

### 3.4 Management of peri-device leak events

A 64-year-old male patient, who stopped his medication after perceiving symptom improvement and had insufficient understanding of DOACs, was unwilling to take any medication long-term. Consequently, he underwent a LAAC with ablation procedure after preoperative assessment. During the procedure, a DSA of the LAA identified a “chicken wing” morphology with an opening diameter of 17 mm and a depth of 21 mm. A 21-mm WATCHMAN device was precisely placed in the LAA, with no residual leaks detected, and the compression ratio ranged from 15% to 20%, ensuring the device was appropriately secure. Post-discharge, the patient was put on a 12-week regimen of rivaroxaban (20 mg daily) and aspirin (100 mg daily) for anticoagulation and antiplatelet therapy. On the 50th day after the procedure, during a routine follow-up, no ischemic stroke, TIA, or bleeding complications were reported. However, a CCTA scan revealed a 5 mm PDL at the lower edge of the device. After ruling out issues related to device size and compression ratio during the procedure, it was speculated that the PDL might be due to incomplete endothelialization around the device. The patient was advised to continue with rivaroxaban and aspirin and undergo regular follow-ups. A CCTA re-examination 2 months later showed that the PDL had reduced to 1.6 mm, and no adverse events were reported in subsequent follow-ups.

### 3.5 Management of systemic embolism events

**Figure 6:**
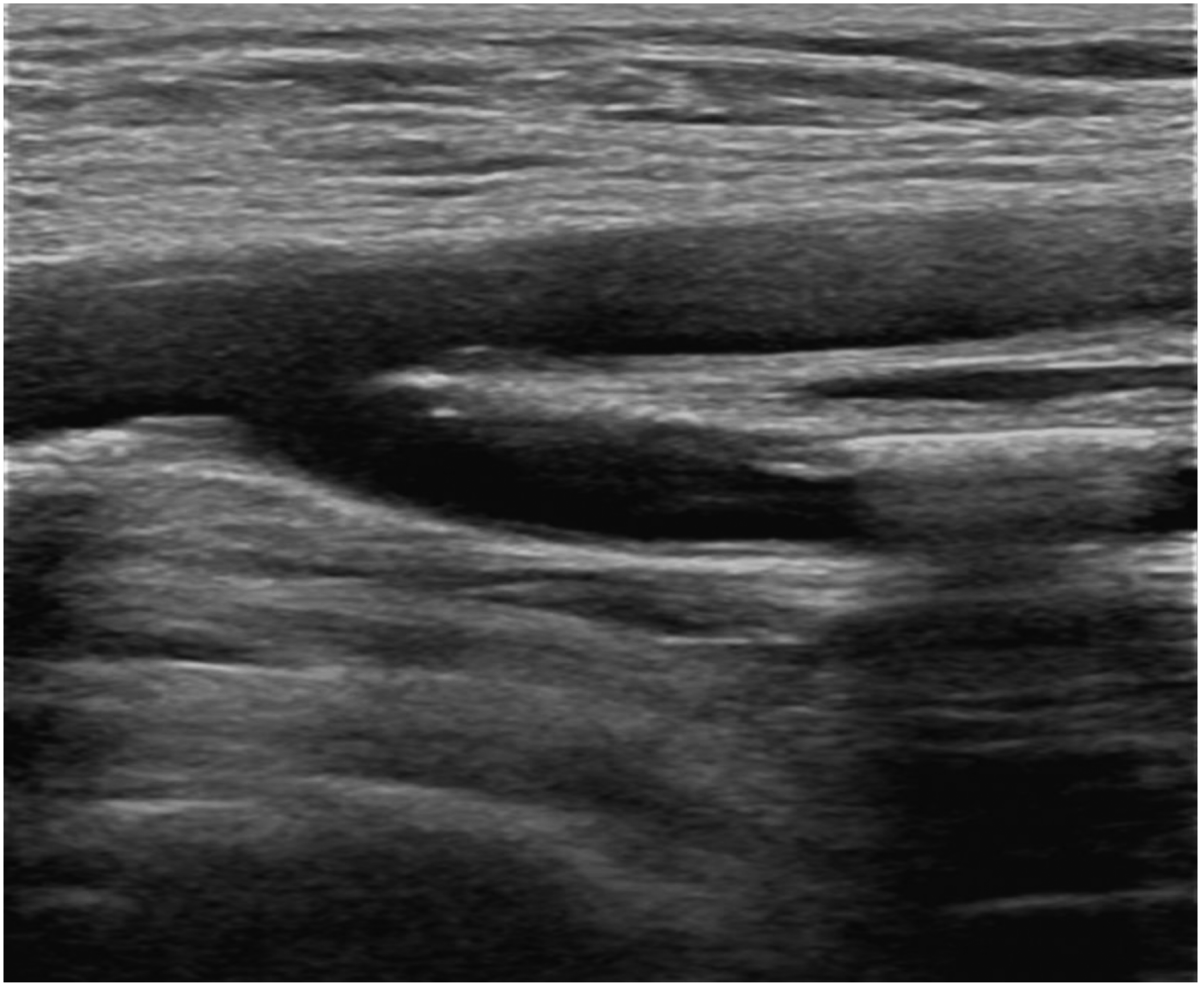
Lower extremity vascular ultrasound suggests lower extremity arterial thrombosis leading to arterial lumen occlusion.

A 76-year-old female patient consulted at our center for ischemic stroke treatment, where she was diagnosed with AF following a comprehensive evaluation. The patient had a HAS-BLED score of 6 (Hypertension, Abnormal liver function, Stroke, Labile INR, Elderly, long-term use of NSAIDs) and also had cognitive impairment. Therefore, we performed the LAAC procedure. After ruling out any contraindications, LAAC was performed as a stroke preventative measure. Post femoral vein puncture, 5,500 units of heparin were administered, and ACT was monitored at 320 s after 5 min. Subsequent septal puncture and LAA DSA revealed a “Windsock” morphology. A 21-mm WATCHMAN device was successfully placed within the LAA, achieving a compression ratio of 15%–20%. Upon discharge, the patient commenced antiplatelet therapy with clopidogrel (75 mg daily) and aspirin (100 mg daily). Follow-up evaluations showed the occluder was well-positioned with no PDL, and the patient experienced no ischemic stroke, TIA, or hemorrhagic complications. However, 6 months post-procedure, the patient presented with leg pain after walking. Lower extremity vascular ultrasound revealed occlusion in the left anterior tibial and foot arteries (Figure 6), with elevated D-dimer levels at 3.51 µg/ml. This raised concerns of a potential systemic embolic event following LAAC. Investigation into the patient's medication adherence revealed she had been taking only aspirin postoperatively. We recommended adding rivaroxaban (20 mg daily) to her regimen for enhanced anticoagulation and antiplatelet effect. This adjusted treatment approach led to no further adverse events in subsequent follow-ups.

### 3.6 Management of bleeding events

A 67-year-old female patient, who enjoys sports and was unwilling to take DOACs, underwent LAAC with the placement of a 23-mm WATCHMAN device, achieving a compression ratio of 15%–20%. Postoperatively, the patient was prescribed rivaroxaban (20 mg daily) and aspirin (100 mg daily) to maintain anticoagulation and antiplatelet therapy. On the third day after procedure, the patient reported reddish urine, and a comprehensive examination revealed significant hematuria with a red blood cell count of 4.41 × 10^12^/L and hemoglobin level of 134 g/L. The ultrasound of the urinary system showed no abnormalities. Given the potential for urinary bleeding complications linked to the anticoagulant therapy post-LAAC, the patient's medication regimen was immediately revised. Aspirin and rivaroxaban were temporarily discontinued, and she was switched to enoxaparin (0.8 ml twice daily) for anticoagulation, accompanied by bladder irrigation. These measures led to the resolution of hematuria by the 11th postoperative day. Upon discharge, the patient resumed anticoagulant and antiplatelet therapy with rivaroxaban (15 mg daily) and aspirin (100 mg daily) for 12 weeks. No complications detected on follow-up.

### 3.7 Management of device-related complications events

**Figure 7:**
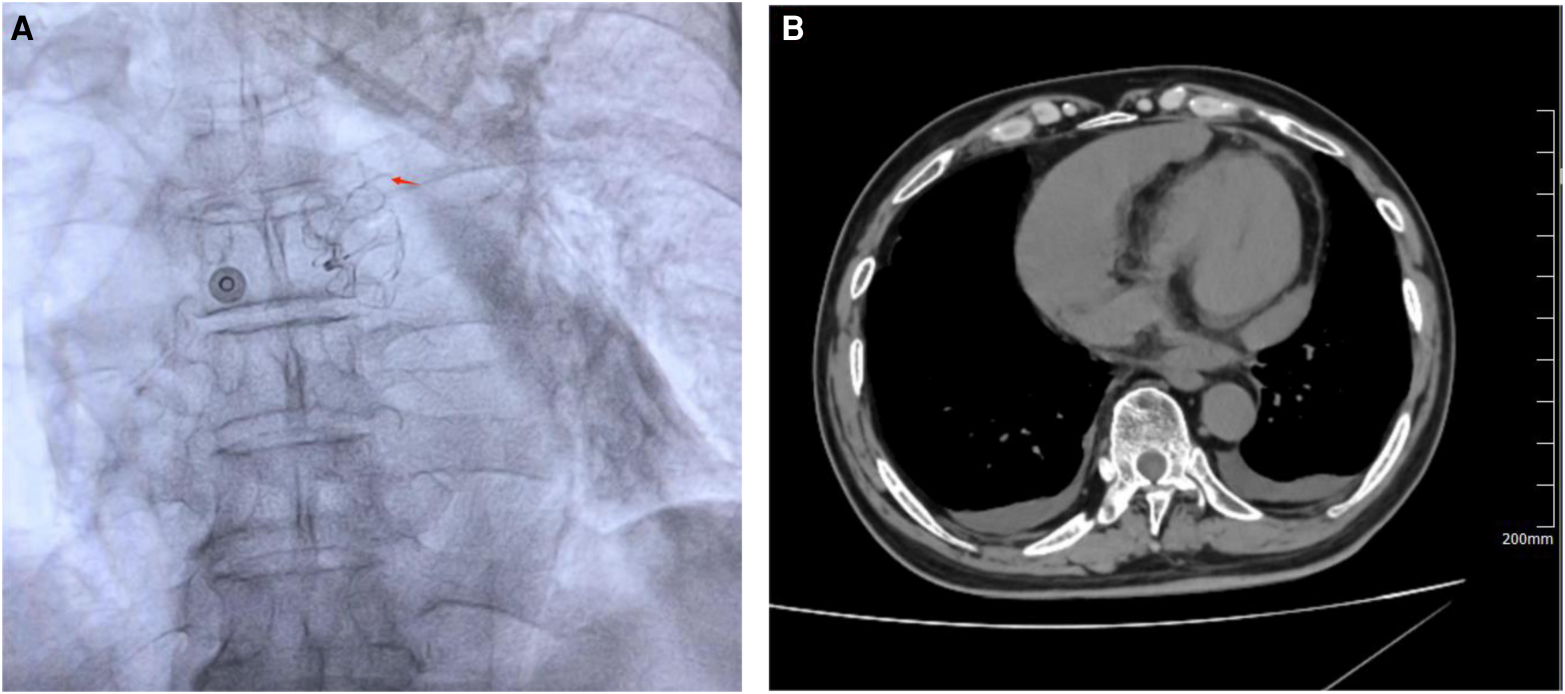
(**A**) DSA revealed that a strut frame of the implanted occluder had dislodged. (**B**) CCTA revealed a large pericardial effusion in the pericardial cavity. Red arrow indicates dislodged occluder strut frame.

A 75-year-old male patient, who works outdoors and is unable to take DOACs long-term due to work requirements, underwent the LAAC procedure after evaluation. A 33-mm WATCHMAN device was successfully implanted in the LAA. DSA confirmed that the device was properly positioned without any anomalies. On the second day post-procedure, the patient reported chest discomfort and shortness of breath, coughing frothy sputum, and a sudden drop in blood pressure to 70/40 mmHg, indicating a possible postoperative pericardial effusion. An urgent pericardiocentesis was performed, extracting 450 ml of sanguineous fluid. Further investigation into the sudden bleeding revealed that a strut frame of the implanted occluder had dislodged (Figure 7A), causing damage to the LAA and leading to the bleeding. After draining the fluid, continuous drainage was conducted over the third and fourth postoperative days, totaling an additional 100 ml of fluid. Upon stabilization, the patient was discharged and commenced on oral rivaroxaban (15 mg daily) and aspirin (100 mg daily) for anticoagulation and antiplatelet therapy. One month after discharge, the patient again experienced chest discomfort and shortness of breath. A detailed echocardiogram and CCTA indicated a significant pericardial effusion, prompting another urgent pericardiocentesis that removed 260 ml of sanguineous fluid (Figure 7B). Continuous pericardial fluid drainage was maintained for 20 days, after which no further drainage was needed. The patient's vital signs stabilized and he was discharged. Post-discharge, the patient continued on rivaroxaban for 12 weeks, after which he switched to clopidogrel (75 mg daily) for another 12 weeks before discontinuing the medication. No complications detected on follow-up.

## 4 Discussion

This study outlines the effective management of emergent complications during the LAAC procedure. We reviewed 11 cases, each with different complications and their successful resolutions. Three patients experienced intraoperative thrombotic events, managed through thrombus aspiration and thrombolysis. One patient discontinued aspirin post-LAAC, leading to a suspected DRT, which resolved after resuming dual therapy. Three cases of pericardial tamponade were successfully managed with drainage and occluder placement. One patient had a PDL, which decreased with continued medication. Another developed leg pain from arterial occlusion after stopping clopidogrel, resolved by adding rivaroxaban. A patient with hematuria postoperatively was managed with enoxaparin and bladder irrigation. Lastly, a patient with pericardial effusion due to a dislodged device strut required pericardiocentesis and further stabilization. These cases highlight the need for prompt and individualized management strategies during LAAC.

### 4.1 Intra-operative device thrombosis

Thrombosis ranks among the prevalent complications of the LAAC procedure. Several factors commonly precipitate intraoperative thrombosis, including: (1) absence or insufficiency of preoperative anti-coagulation; (2) extended operative durations, coupled with inadequate ACT monitoring and heparin supplementation; (3) insufficient preoperative heparinized saline flushing of catheters and guidewires; and (4) inherent patient conditions such as a hypercoagulable state or heparin resistance. In our facility, we encountered three instances of intraoperative thrombosis during LAAC. One case involved a patient diagnosed with AF, who received immediate anti-coagulation therapy with DOAC (rivaroxaban 20 mg q.d.). The LAAC procedure was initiated six days post-anticoagulation. Despite the intraoperative administration of 6,000 u of heparin, an increasing presence of flocculent material, indicative of thrombosis, was observed at the catheter's head end. Initial ACT readings were 160 s, increasing marginally to 180 s 5 min after an additional 2,000 u of heparin was administered. Given these dynamics, we hypothesized heparin resistance. Addressing this, we administered another 2,000 u of heparin, after which ACT readings rose to 240 s within 5 min, and the flocculent material substantially receded, corroborating the efficacy of our intervention. Moreover, we recognize that inadequate preoperative anti-coagulation may have also contributed to this patient's intraoperative thrombosis. This realization underscores the clinical imperative of ensuring adequate anti-coagulation before scheduling an LAAC procedure, significantly mitigating the risk of intraoperative thrombosis. Additionally, protracted surgery duration emerges as a critical risk factor for intraoperative thrombosis, necessitating diligent surgical efficiency and vigilant patient monitoring. In another instance, we executed a LAAC with ablation procedure. Research indicates that while the LAAC with ablation strategy can enhance left atrial structure and restore sinus rhythm in AF patients, it also carries potential drawbacks. One significant complication is intraoperative thrombosis. The extended procedure duration and prolonged catheter placement in the vessel trigger clotting factor activation and platelet adhesion. Without timely ACT monitoring and appropriate heparin supplementation, the risk of thrombosis escalates (12). Thus, the inclusion of a CA procedure, extending total operative time, emerges as an additional thrombosis risk factor during LAAC. Moreover, acute atrial myocardial injury from RFCA can lead to postoperative troponin release and tissue edema, heightening thrombus formation risks in the left atrial or on the device surface. In cases of thrombus development, we advocate for proactive thrombolysis. Importantly, prior to thrombolysis, our protocol involves positioning a CPS at the patient's bilateral internal carotid arteries. Although approved for transcatheter aortic valve replacement (TAVR), no consensus or guidelines endorse CPS use in LAAC procedures (13). Perioperative stroke is a devastating complication, with reported rates of perioperative cerebrovascular events (including transient ischemic attacks and embolic strokes) ranging from 0.8% to 1.8% (14, 15). During thrombolysis after intraoperative thrombosis, ruptured and dislodged debris may migrate into cerebral arteries, inducing new ischemic injuries. Although some studies have shown that embolism resulting from this approach is sub-clinical, it can also cause some neurocognitive dysfunction in patients. There are limited strategies to preempt medically induced cerebral embolic events, thus the presence of a CPS is particularly important (16). In a case of intraoperative thrombosis, we employed a CPS, followed by thrombolysis. Post-thrombolysis examination of the CPS revealed a linear white thrombus within the right internal carotid artery's filter, underscoring the CPS's critical function during intraoperative thrombolysis subsequent to LAAC thrombosis. However, the CPS's shielding was not absolute. Its dual arterial filters failed to safeguard the left vertebral and subclavian arteries, along with the descending aorta and coronary arteries. In this case of intraoperative thrombosis, despite bilateral CPS deployment to protect the internal carotid artery prior to thrombolysis, intraoperatively dislodged thrombus passed through the vertebral artery resulting in a transient cerebral ischaemic event. Such events post-CPS placement are seldom highlighted in literature. Hence, the cerebral protective efficacy of CPS should not be overestimated. Future iterations of CPS devices should incorporate considerations for comprehensive cerebral defense intraoperatively to mitigate cerebral embolic incidents. Such advancements could significantly enhance postoperative outcomes and patients' quality of life. In addition, our study emphatically underscores the critical role of TEE within the context of the LAAC procedure. TEE serves a dual purpose: it facilitates the operator's guidance during the septal puncture process and proves instrumental in the prompt detection of intraoperative thrombosis. This timely identification allows for immediate intervention, forestalling the onset of medically induced thromboembolic events.

### 4.2 Device-related thrombosis

DRT is a significant complication following LAAC, occurring in approximately 2.0%–7.2% of patients. Studies suggest that DRT increases the risk of stroke and systemic embolic events, potentially negating the benefits of LAAC and impacting patients both physically and psychologically (17, 18). Effective prevention of DRT is thus critical during postoperative care. Several factors contribute to the incidence of postoperative DRT: (1) Patient-Related Factors: Advanced age, previous TIA or strokes, existing cardiac diseases, a high CHA₂DS₂-VASc score, and spontaneous echocardiographic contrast in the left atrium are predictive of DRT; (2) Device Implantation Issues: Over-deployment of the occluder, large occluder size, and PDL around the device are linked to DRT and (3) Postoperative Management: Inadequate standardization in LAAC procedures and non-adherence to antithrombotic medication regimens are common issues (19–21). Following LAAC, endothelialization of the occluder requires time, during which the device remains exposed to bloodstream elements, increasing thrombus formation risk. It is crucial to adhere to postoperative anticoagulation and antiplatelet protocols (18, 22).

Current guidelines advocate for 3 months of direct oral anticoagulation followed by 6 months of dual antiplatelet therapy and lifelong aspirin use thereafter, contingent upon the exclusion of DRT (23). Despite these recommendations, non-compliance with medication regimens has led to DRT events at our center. For example, one patient discontinued his combined aspirin and rivaroxaban therapy 1 month post-procedure, resulting in DRT. The patients resumed dual therapy on a 3-month course after being informed of the risks, resulting in no subsequent DRT events. It is important to note that DRT can be classified into early and late occurrences, each with different causes and management strategies. Early DRT typically occurs within a few months post-procedure and is mainly related to technical factors during surgery, incomplete endothelialization of the device, or individual patient differences. Common causes of early DRT include incomplete embedding of the device in the appendage, residual leaks, and local damage caused by intraoperative maneuvers. To prevent early DRT, strict anticoagulation therapy, such as the use of warfarin or DOACs, is usually administered in the initial postoperative period to prevent thrombus formation. Late DRT can occur months to years after the procedure and is often associated with the patient's overall health condition. For example, chronic inflammatory states, prothrombotic conditions, or new-onset arrhythmias can lead to late DRT. Late DRT indicates the need for long-term follow-up and comprehensive evaluation of patients, particularly focusing on underlying systemic diseases and chronic disease management. Additionally, the occurrence of late DRT is closely related to patient compliance, interruptions in long-term anticoagulation therapy, and improper management of underlying conditions. Effective management of late DRT requires long-term imaging follow-up and regular clinical evaluations post-surgery. Furthermore, individualized anticoagulation therapy plans and patient education are crucial for preventing late DRT (4, 24, 25).

While LAAC is an effective stroke prevention strategy in patients with AF, managing DRT remains a challenge. Our experience underscores the importance of stringent postoperative management and patient education regarding antithrombotic medication to prevent DRT.

### 4.3 Pericardial effusion/tamponade

The occurrence of pericardial effusion and pericardial tamponade is one of the severe complications within LAAC, often attributable to surgical maneuvers. Previous studies have shown that the incidence rates of pericardial effusion and cardiac tamponade are 2.9% and 0.5%, respectively (26). Typical etiologies include: (1) atrial perforation during septal puncture by the needle or sheath. (2). LAA puncture due to improper guide-wire or catheter manipulation. (3). Atrial appendage puncture induced by device placement (4). LAA laceration during device retrieval. (5). LAA tearing from excessive retraction force applied to the device. When pericardial effusion arises, prompt and adept management becomes vital, both for saving lives and enhancing patient prognosis, thereby challenging our professional competence significantly. Among the three pericardial effusion/tamponade incidents at our center, one was complicated by the patient's “pendulous heart” morphology, which precipitated a high septal puncture position and procedural difficulty. This patient developed pericardial effusion following deep LAA catheter penetration. The operator introduced a 10-mm atrial septal defect occluder into the LAA to halt bleeding, circumventing a perilous situation and the necessity for more invasive surgical LAA access. This technique, sparsely documented in the literature, serves as a valuable reference for handling such emergent scenarios, especially for LAA with shallow, broad bases. Crucially, this strategy mandates retaining the guide-wire or catheter within the pericardium post-penetration to ease device delivery. Postoperative assessments confirmed the occluder's optimal positioning, with no subsequent displacement or residual leakage noted. In a case we have encountered, but not mentioned in this report. A patient undergoing a LAAC with ablation procedure experienced a LAA puncture during device placement post-successful RFCA, leading to sudden pericardial effusion. The severity of the bleeding necessitated an emergency open-heart LAA suture by a cardiac surgeon, resulting in a 19-day hospitalization. Unlike the prior incident, this patient's recovery was protracted, and the experience was psychologically taxing due to the invasive nature of an open-heart suture.

In two other instances of pericardial tamponade during the procedure, we adopted a provisional measure to manage the complication while preparing for potential open-heart surgery. We temporarily released the device post-tamponade onset to seal the breach and curtail further bleeding. Fortuitously, we observed that bleeding ceased following the device's release. We postulate that this cessation may have resulted from intraoperative maneuvers that inadvertently scratched the atrial appendage, with the device's release fortuitously sealing the breach. This strategy is contingent on specific circumstances, namely minor bleeding and a smaller breach, and mandates vigilant post-release patient monitoring. In cases of significant bleeding, our inclination is toward an emergency surgical open-chest suture. Intriguingly, all three cases of pericardial effusion and tamponade at our center involved female patients. While our current study does not extrapolate significance from this pattern, prior research offers compelling insights. Schmidt et al. postulated that the female gender might predispose patients to pericardial effusion during LAAC. Concurrently, Yang et al. identified correlations among female gender, paroxysmal atrial fibrillation, sinus rhythm alterations, procedural timing, and the onset of perioperative pericardial effusion in a cohort of 624 LAAC patients (27, 28). Furthermore, a comprehensive meta-analysis encompassing 19 studies revealed a higher propensity for major procedural complications among women undergoing RFCA for atrial fibrillation. These complications include pericardial effusion or tamponade and significant hemorrhage necessitating blood transfusion, diverging noticeably from their male counterparts. This discrepancy may originate from inherent variances in atrial morphology, structure, and function between genders. Additionally, female LAAC candidates often present with advanced age, an extended duration of atrial fibrillation, and a higher burden of chronic diseases (29). Consequently, heightened caution is advisable during patient assessment and LAAC indication evaluation, particularly for elderly women and those undergoing the LAAC with ablation procedure. Proactive engagement in preoperative dialogues and meticulous preparatory measures are essential to anticipate and strategically plan for unforeseen intraoperative occurrences.

### 4.4 Peri-device leak

In this case series, we observed a 64-year-old male patient who experienced PDL following LAAC. Although accurate measurement of the LAA and the selection of an appropriately sized occluder typically result in low PDL incidence, such complications can still pose significant challenges in patient management and treatment. The clinical significance of PDL is currently debated in research. Some studies suggest that PDL might diminish the benefits of LAAC for AF patients by potentially increasing the risk of DRT. The reported incidence of PDL varies from 0% to 63%, depending on the type of LAAC device used as well as the frequency and method of monitoring (30). For instance, Bai et al. identified PDL as a critical factor influencing DRT occurrence in a cohort of 319 patients post-LAAC, a finding corroborated by other researchers (19, 31, 32). Afzal et al., in a retrospective analysis of 1,039 LAAC patients, noted a higher incidence of TIA and strokes among patients with residual leaks compared to those without (33). Conversely, some studies argue that PDL does not significantly impact clinical outcomes for AF patients (34, 35). The mechanisms leading to PDL include off-axis placement, inadequate sealing due to non-perpendicular alignment between the LAA landing zone and the occluder flaps, gaps around the occluder due to insufficient expansion at the LAA landing zone, incomplete endothelialization, and selection of an inappropriate occluder size or inadequate compression ratio during procedure. Correct occluder selection and placement are crucial for minimizing PDL (36). In this instance, a 21-mm Watchman device was selected based on the LAA's anatomical characteristics, with a compression ratio of 15%–20% suggesting optimal contact and fixation. Nevertheless, a 5 mm PDL was observed in a follow-up CCTA, likely due to incomplete endothelialization. The patient was advised to maintain antithrombotic therapy with rivaroxaban and aspirin. Subsequent imaging showed a reduction in the leak size to 1.6 mm, emphasizing the need for ongoing antithrombotic therapy to manage PDL and prevent thrombotic complications.

### 4.5 Systemic embolism

Although LAAC procedures are effective in preventing thromboembolic events, they still pose a risk of systemic embolism. Defined by the Munich consensus document, post-LAAC systemic embolism involves acute vascular insufficiency or occlusion of extremities or non-CNS organs, substantiated by clinical, imaging, surgical, or autopsy evidence of arterial occlusion without other probable causes. Research indicates that systemic embolism following LAAC occurs relatively infrequently, with incidences ranging from 0.24% to 0.29% (37, 38). Several factors contribute to systemic embolism, including inadequate interaction between the occluder and cardiac tissue, insufficient postoperative antithrombotic therapy, and individual conditions like increased blood viscosity. This case of a 76-year-old female, who underwent LAAC to prevent stroke associated with AF and later presented with leg pain and elevated D-dimer indicating distal arterial embolism, exemplifies the potential risks. It highlights the need for comprehensive management strategies following LAAC. The patient's reliance solely on aspirin for postoperative antiplatelet therapy may have been insufficient to prevent thrombus formation. A more robust early postoperative management, integrating both antiplatelet and oral anticoagulant therapies, is recommended based on risk assessments. Her treatment regimen, which later included aspirin combined with rivaroxaban, underscores the importance of personalized treatment plans. Moreover, regular monitoring of vascular status and thrombotic markers, such as D-dimer, is essential for the early detection and prevention of thrombotic complications.

This case underlines the risk of systemic embolism post-LAAC and the critical need for tailored antithrombotic strategies in postoperative care. Ongoing clinical and imaging follow-ups, coupled with individualized risk assessment and treatment adjustments, are vital to optimize treatment outcomes and minimize complications. Future research should focus on refining antithrombotic therapy to balance the risks of embolism and bleeding and on developing strategies to monitor and prevent systemic embolic events effectively.

### 4.6 Bleeding

Anticoagulant and antiplatelet therapies are essential for reducing postoperative thrombosis after LAAC, yet they elevate the risk of bleeding. In this instance, a 67-year-old female developed urinary system bleeding complications post-LAAC, following anticoagulant and antiplatelet treatments. This scenario underscores the necessity of balancing the benefits of anticoagulation with the risks of bleeding and devising appropriate management strategies. Bleeding is a commonly observed complication post-LAAC, especially in elderly patients or those with predispositions to bleeding. The EWOLUTION trial found that the incidence of bleeding post-LAAC is 2.7% (39). Upon detection of bleeding, the medical team implemented judicious measures, including the temporary cessation of oral anticoagulants and antiplatelets, transitioning to enoxaparin for continued anticoagulation. This method provided greater flexibility in managing anticoagulation intensity and facilitated the cautious reintroduction of oral anticoagulants once the bleeding was under control. The patient's adherence to a modified anticoagulation regimen after stabilizing the bleeding highlights the critical balance between minimizing bleeding risks and preventing thrombotic complications. In clinical practice, it is imperative to tailor anticoagulation strategies to individual patient risk profiles. This case illustrates the importance of vigilant monitoring for bleeding in patients on anticoagulation therapy and the necessity for adaptable treatment plans. Furthermore, it demonstrates that prompt and appropriate interventions can effectively manage complications and avert severe outcomes. Physicians must diligently evaluate patients at heightened risk of bleeding and closely monitor them throughout their treatment.

### 4.7 Device-related complications

This case highlights the complex management of a 75-year-old male who experienced significant postoperative complications following LAAC with a 33 mm Watchman device. The initial presentation of chest discomfort, frothy sputum, and hypotension suggested a cardiovascular etiology, which was confirmed to be pericardial effusion by emergent pericardiocentesis, extracting 450 ml of sanguineous fluid. Further investigations revealed a mechanical complication with the device—a dislodged strut frame—which inflicted damage on the left atrial appendage, leading to bleeding. The displacement of the Watchman device's strut frame underscores the potential mechanical failures that can result in severe clinical outcomes such as cardiac tamponade. Although the incidence of such complications is low, they underscore the necessity for precise device deployment and the potential for structural device failures post-implantation. For the pericardial effusions that did occur, our management strategy included repeated pericardiocentesis and prolonged drainage, suggesting that bleeding complications from mechanical trauma caused by the device persisted. In this context, it is necessary to discuss postoperative monitoring and management protocols for LAAC, especially for patients who present with early signs of postoperative discomfort. Post-discharge, the patient was managed with rivaroxaban and aspirin to mitigate the risk of thromboembolic events. The re-presentation of symptoms and subsequent large pericardial effusion required further intervention, highlighting the delicate balance between preventing thrombosis and exacerbating bleeding risks in post-LAAC patients. The transition from rivaroxaban to clopidogrel and the eventual cessation of antiplatelet therapy illustrate a tailored approach to managing individual patient risks and responses. Stable vital signs and cessation of drainage 20 days post-second intervention allowed for discharge, and subsequent follow-up did not reveal any ischemic or bleeding complications, suggesting effective resolution of the immediate postoperative issues. This case emphasizes the importance of vigilant postoperative monitoring and readiness to address complications through interventional and pharmacologic strategies. For similar cases, we recommend (1) Enhanced Monitoring: Patients receiving LAAC, especially those with complex cardiovascular histories, should be monitored closely for signs of pericardial effusion or device malposition; (2) Readiness for Intervention: Facilities performing LAAC should be equipped and prepared to perform emergency interventions such as pericardiocentesis and (3) Tailored Pharmacotherapy: Anticoagulant and antiplatelet regimens should be personalized based on ongoing assessments of bleeding and thrombotic risks post-LAAC. This case underlines the importance of comprehensive perioperative planning, meticulous procedural execution, and the need for proactive management strategies to address both anticipated and unforeseen complications following LAAC. Further research may focus on refining antithrombotic protocols and enhancing device design to minimize the risk of structural failures and improve patient outcomes.

### 4.8 Device embolization

Device embolization is a rare but serious complication of LAAC, recognized for its potentially life-threatening effects on patient safety. Clinical studies, including PREVAIL and PROTECT AF, report its incidence at approximately 0.24% (40, 41). Factors contributing to device embolization include: (1) Device Size Mismatch: Inadequately sized occluders may dislocate if they do not fit the left atrial appendage properly; (2) Operational Technique: The expertise and technique of the surgeon can critically influence the stability of the device; (3) Anatomical Specificity: Variability in the size and shape of the LAA can make stable device fixation challenging and (4) Effective management of device embolization is crucial and requires immediate actions to improve the success rate of the procedure and ensure patient safety. Standard management techniques involve repositioning or retrieving the device, utilizing various techniques such as transseptal from the left atrium, transarterial from the aorta, or from the left ventricle, as demonstrated by experts like Shinwan (42). Prompt detection of device embolization is essential, typically involving postoperative imaging studies like echocardiograms or CCTA to identify any signs of displacement. The emergence of new cardiac symptoms such as arrhythmias or hemodynamic changes post-procedure should also prompt consideration of possible device embolization. Minimizing the risk of device embolization necessitates thorough preoperative evaluations and precise device selection. Additionally, employing an experienced procedure team and utilizing real-time imaging during the procedure can significantly reduce the risk of embolization. These strategies emphasize the necessity of meticulous preparation, skilled execution, and swift intervention in the management of complications associated with LAAC procedures. By adhering to these practices, healthcare providers can enhance outcomes for patients undergoing LAAC.

### 4.9 Pulmonary erosion/perforation

Pulmonary erosion or perforation is a rare but serious complication associated with LAAC devices such as the Watchman. This complication arises when the device or a part of it erodes through the heart or atrial wall into adjacent structures such as the lung or pulmonary veins. The proximity of the left atrial appendage to the pulmonary artery and lung makes this region particularly susceptible to such complications. Patients with pulmonary erosion or perforation may present with symptoms ranging from unexplained dyspnea and chest pain to more severe signs such as hemoptysis and hypotension, which could indicate a life-threatening situation. Diagnosis often involves imaging studies such as CT or echocardiography, which can provide detailed information about the location and extent of the erosion or perforation. Prevention starts with careful patient selection, precise imaging before the procedure to ensure appropriate sizing and positioning of the device, and meticulous surgical technique. It is crucial that the deploying physician ensures the device does not exert excessive pressure on the atrial wall and is well away from the pulmonary structures. Once this complication occurs, prompt and effective management is crucial, and currently effective treatment strategies include (1) Immediate Response: On identification of a pulmonary complication, stabilizing the patient is paramount. This involves managing any acute symptoms, ensuring cardiovascular stability, and providing supportive care; (2) Surgical Intervention: In cases where there is significant erosion or risk of further complications, surgical repair or removal of the device may be necessary. This could involve procedures such as pericardial patch repair or even partial lobectomy depending on the location and severity of the erosion; (3) Long-term Management: Long-term follow-up with regular imaging is advisable to monitor for any late complications or recurrences. In cases where the device remains *in situ*, close monitoring for signs of infection or device migration is critical and (4) Pharmacological Therapy: Depending on the situation, management may also include the use of antibiotics to prevent infection, and adjustments in anticoagulant therapy to manage bleeding risks without increasing the risk of thrombosis.

While pulmonary erosion or perforation following LAAC is rare, recognizing potential risk factors and early symptoms can be crucial for timely intervention. The development of such complications necessitates a multidisciplinary approach involving cardiologists, cardiothoracic surgeons, and radiologists to ensure optimal patient outcomes. Further research into the prevention and management of this complication is needed to refine guidelines and improve safety for patients undergoing LAAC.

## 5 Limitation

There may be some limitations to our study. First, the results are based on data from a single center, which may limit their applicability to other settings. Differences in patient demographics, clinical practices, and complication management strategies between centers could affect the study results. Second, the selection of 11 out of the total 24 cases with complications was aimed at ensuring a comprehensive and representative analysis. However, for the remaining complications, some patients did not have follow-up data or lacked complete clinical information, which restricted our ability to include them in the analysis. Third, follow-up could be extended beyond the current duration of 1 year to better capture long-term outcomes and late complications. Longer follow-up is necessary to fully understand the durability of management strategies and the long-term safety of the procedure. Fourth, the lack of randomization in this case series increases the risk of selection bias. Patients included in the study may have specific characteristics that make them more or less likely to develop complications, affecting the validity of the study. Additionally, the retrospective design may introduce reporting bias, as complications could have been under-reported, potentially leading to an underestimation of the true incidence of complications.

## 6 Conclusion

The study underscores the significance of immediate and targeted interventions for procedural-related complications in LAAC and the LAAC with ablation procedure for AF. Our findings reveal that with the prompt recognition and management of complications such as intraoperative thrombosis and pericardial effusion or tamponade, the procedural success rate can be significantly enhanced, thereby reducing postoperative mortality and morbidity. The strategic deployment of devices in cases of bleeding, has proven effective in ensuring patient safety and improving outcomes. This proactive approach to complication management not only ensures the safe discharge of patients but also contributes to the long-term prevention of stroke without the occurrence of adverse events during follow-up. Our experience and management strategies serve as a testament to the viability and safety of LAAC and the LAAC with ablation procedure as a prophylactic strategy for patients with non-valvular AF who are ineligible for anti-coagulation therapy, thereby reinforcing the procedure's role in comprehensive AF management.

## Data Availability

The original contributions presented in the study are included in the article/Supplementary Material, further inquiries can be directed to the corresponding author.

## References

[1] Schnabel RB Yin X Gona P Larson MG Beiser AS McManus DD 50 year trends in atrial fibrillation prevalence, incidence, risk factors, and mortality in the Framingham heart study: a cohort study. Lancet. (2015) 386:154–62. 10.1016/S0140-6736(14)61774-8 25960110 PMC4553037

[2] Wang Y Bajorek B. New oral anticoagulants in practice: pharmacological and practical considerations. Am J Cardiovasc Drugs. (2014) 14:175–89. 10.1007/s40256-013-0061-0 24452600

[3] Lee SR Choi EK Kwon S Han KD Jung JH Cha MJ Effectiveness and safety of contemporary oral anticoagulants among Asians with nonvalvular atrial fibrillation. Stroke. (2019) 50:2245–9. 10.1161/STROKEAHA.119.025536 31208303

[4] Osmancik P Herman D Neuzil P Hala P Taborsky M Kala P Left atrial appendage closure versus direct oral anticoagulants in high-risk patients with atrial fibrillation. J Am Coll Cardiol. (2020) 75:3122–35. 10.1016/j.jacc.2020.04.067 32586585

[5] Camm AJ Lip GY De Caterina R Savelieva I Atar D Hohnloser SH 2012 focused update of the ESC guidelines for the management of atrial fibrillation: an update of the 2010 ESC guidelines for the management of atrial fibrillation. Developed with the special contribution of the European heart rhythm association. Eur Heart J. (2012) 33:2719–47. 10.1093/eurheartj/ehs253 22922413

[6] Holmes DR Jr Kar S Price MJ Whisenant B Sievert H Doshi SK Prospective randomized evaluation of the watchman left atrial appendage closure device in patients with atrial fibrillation versus long-term warfarin therapy: the PREVAIL trial. J Am Coll Cardiol. (2014) 64:1–12. 10.1016/j.jacc.2014.04.029 24998121

[7] He B Jiang LS Hao ZY Wang H Miao YT. Combination of ablation and left atrial appendage closure as “one-stop” procedure in the treatment of atrial fibrillation: current status and future perspective. Pacing Clin Electrophysiol. (2021) 44:1259–66. 10.1111/pace.14201 33629763 PMC8359309

[8] Reddy VY Gibson DN Kar S O’Neill W Doshi SK Horton RP Post-approval U.S. experience with left atrial appendage closure for stroke prevention in atrial fibrillation. J Am Coll Cardiol. (2017) 69:253–61. 10.1016/j.jacc.2016.10.010 27816552

[9] Yang L Zhang X Jin Q Kong D Zhang Y Li M Pericardial effusion during the perioperative period for left atrial appendage closure. Front Cardiovasc Med. (2021) 8:678460. 10.3389/fcvm.2021.678460 34409074 PMC8365031

[10] Tzikas A Holmes DR Jr Gafoor S Ruiz CE Blomström-Lundqvist C Diener HC Percutaneous left atrial appendage occlusion: the Munich consensus document on definitions, endpoints, and data collection requirements for clinical studies. Europace. (2017) 19:4–15. 10.1093/europace/euw141 27540038 PMC5841559

[11] Rose DZ Chang JY Yi X Kip K Lu Y Hilker NC Direct oral anticoagulant failures in atrial fibrillation with stroke: retrospective admission analysis and novel classification system. Neurohospitalist. (2023) 13:256–65. 10.1177/19418744231161390 37441203 PMC10334065

[12] Packer M. Effect of catheter ablation on pre-existing abnormalities of left atrial systolic, diastolic, and neurohormonal functions in patients with chronic heart failure and atrial fibrillation. Eur Heart J. (2019) 40:1873–9. 10.1093/eurheartj/ehz284 31081029 PMC6568203

[13] Rogers T Alraies MC Torguson R Waksman R. Overview of the 2017 US food and drug administration circulatory system devices panel meeting on the sentinel cerebral protection system. Am Heart J. (2017) 192:113–9. 10.1016/j.ahj.2017.06.007 28938957

[14] Kuck KH Schaumann A Eckardt L Willems S Ventura R Delacrétaz E Catheter ablation of stable ventricular tachycardia before defibrillator implantation in patients with coronary heart disease (VTACH): a multicentre randomised controlled trial. Lancet. (2010) 375:31–40. 10.1016/S0140-6736(09)61755-4 20109864

[15] Bohnen M Stevenson WG Tedrow UB Michaud GF John RM Epstein LM Incidence and predictors of major complications from contemporary catheter ablation to treat cardiac arrhythmias. Heart Rhythm. (2011) 8:1661–6. 10.1016/j.hrthm.2011.05.017 21699857

[16] Heeger CH Metzner A Schlüter M Rillig A Mathew S Tilz RR Cerebral protection during catheter ablation of ventricular tachycardia in patients with ischemic heart disease. J Am Heart Assoc. (2018) 7:e009005. 10.1161/JAHA.118.009005 29960991 PMC6064920

[17] Black-Maier E Piccini JP Granger CB. Left atrial appendage closure: a therapy uniquely suited for specific populations of patients with atrial fibrillation. J Cardiovasc Electrophysiol. (2019) 30:2968–76. 10.1111/jce.14182 31520437

[18] Fauchier L Cinaud A Brigadeau F Lepillier A Pierre B Abbey S Device-related thrombosis after percutaneous left atrial appendage occlusion for atrial fibrillation. J Am Coll Cardiol. (2018) 71:1528–36. 10.1016/j.jacc.2018.01.076 29622159

[19] Bai Y Xue X Duenninger E Muenzel M Jiang L Keil T Real-world survival data of device-related thrombus following left atrial appendage closure: 4-year experience from a single center. Heart Vessels. (2019) 34:1360–9. 10.1007/s00380-019-01364-7 30820642

[20] Main ML Fan D Reddy VY Holmes DR Gordon NT Coggins TR Assessment of device-related thrombus and associated clinical outcomes with the WATCHMAN left atrial appendage closure device for embolic protection in patients with atrial fibrillation (from the PROTECT-AF trial). Am J Cardiol. (2016) 117:1127–34. 10.1016/j.amjcard.2016.01.039 26993976

[21] Pracon R Bangalore S Dzielinska Z Konka M Kepka C Kruk M Device thrombosis after percutaneous left atrial appendage occlusion is related to patient and procedural characteristics but not to duration of postimplantation dual antiplatelet therapy. Circ Cardiovasc Interv. (2018) 11:e005997. 10.1161/CIRCINTERVENTIONS.117.005997 29463510

[22] Duthoit G Silvain J Marijon E Ducrocq G Lepillier A Frere C Reduced rivaroxaban dose versus dual antiplatelet therapy after left atrial appendage closure: ADRIFT a randomized pilot study. Circ Cardiovasc Interv. (2020) 13:e008481. 10.1161/CIRCINTERVENTIONS.119.008481 32674675

[23] Glikson M Wolff R Hindricks G Mandrola J Camm AJ Lip GYH EHRA/EAPCI expert consensus statement on catheter-based left atrial appendage occlusion—an update. Europace. (2020) 22:184. 10.1093/europace/euz258 31504441

[24] Price MJ. Device-related thrombus after transcatheter left atrial appendage closure. JACC Cardiovasc Interv. (2019) 12:1015–7. 10.1016/j.jcin.2019.03.039 31103539

[25] Armouti AO Amin S Rose DZ. Late device-related thrombus associated with occult malignancy years after left atrial appendage closure. J Stroke Cerebrovasc Dis. (2024) 33:107618. 10.1016/j.jstrokecerebrovasdis.2024.107618 38402694

[26] Vuddanda VLK Turagam MK Umale NA Shah Z Lakkireddy DR Bartus K Incidence and causes of in-hospital outcomes and 30-day readmissions after percutaneous left atrial appendage closure: a US nationwide retrospective cohort study using claims data. Heart Rhythm. (2020) 17:374–82. 10.1016/j.hrthm.2019.09.018 31539630

[27] Schmidt B Betts TR Sievert H Bergmann MW Kische S Pokushalov E Incidence of pericardial effusion after left atrial appendage closure: the impact of underlying heart rhythm-data from the EWOLUTION study. J Cardiovasc Electrophysiol. (2018) 29:973–8. 10.1111/jce.13626 29722469

[28] Cheng X Hu Q Gao L Liu J Qin S Zhang D. Sex-related differences in catheter ablation of atrial fibrillation: a systematic review and meta-analysis. Europace. (2019) 21:1509–18. 10.1093/europace/euz179 31281922

[29] Odening KE Deiß S Dilling-Boer D Didenko M Eriksson U Nedios S Mechanisms of sex differences in atrial fibrillation: role of hormones and differences in electrophysiology, structure, function, and remodelling. Europace. (2019) 21:366–76. 10.1093/europace/euy215 30351414

[30] Turagam MK Velagapudi P Kar S Holmes D Reddy VY Refaat MM Cardiovascular therapies targeting left atrial appendage. J Am Coll Cardiol. (2018) 72:448–63. 10.1016/j.jacc.2018.05.048 29954658 PMC8420938

[31] Li W Gao R Zhao J Ren Y Chen G Zhu J Safety and efficacy of different anticoagulation regimens after left atrial appendage occlusion. Ann Palliat Med. (2022) 11:201–9. 10.21037/apm-21-3654 35144411

[32] Sasko B Ritter O Bramlage P Riediger F. Late left atrial appendage closure device displacement and massive thrombus formation: a case report. Eur Heart J Case Rep. (2020) 4:1–5. 10.1093/ehjcr/ytaa014 32352063 PMC7180543

[33] Afzal MR Gabriels JK Jackson GG Chen L Buck B Campbell S Temporal changes and clinical implications of delayed peridevice leak following left atrial appendage closure. JACC Clin Electrophysiol. (2022) 8:15–25. 10.1016/j.jacep.2021.06.018 34454881

[34] Saw J Tzikas A Shakir S Gafoor S Omran H Nielsen-Kudsk JE Incidence and clinical impact of device-associated thrombus and peri-device leak following left atrial appendage closure with the amplatzer cardiac plug. JACC Cardiovasc Interv. (2017) 10:391–9. 10.1016/j.jcin.2016.11.029 28231907

[35] Nguyen A Gallet R Riant E Deux JF Boukantar M Mouillet G Peridevice leak after left atrial appendage closure: incidence, risk factors, and clinical impact. Can J Cardiol. (2019) 35:405–12. 10.1016/j.cjca.2018.12.022 30935631

[36] Saw J Fahmy P DeJong P Lempereur M Spencer R Tsang M Cardiac CT angiography for device surveillance after endovascular left atrial appendage closure. Eur Heart J Cardiovasc Imaging. (2015) 16:1198–206. 10.1093/ehjci/jev067 25851318 PMC4609159

[37] Benjamin EJ Blaha MJ Chiuve SE Cushman M Das SR Deo R Heart disease and stroke statistics-2017 update: a report from the American Heart Association. Circulation. (2017) 135:e146–603. 10.1161/CIR.0000000000000485 28122885 PMC5408160

[38] Khalil F Arora S Killu AM Tripathi B DeSimone CV Egbe A Utilization and procedural adverse outcomes associated with Watchman device implantation. Europace. (2021) 23:247–53. 10.1093/europace/euaa219 32929501

[39] Boersma LV Ince H Kische S Pokushalov E Schmitz T Schmidt B Evaluating real-world clinical outcomes in atrial fibrillation patients receiving the WATCHMAN left atrial appendage closure technology: final 2-year outcome data of the EWOLUTION trial focusing on history of stroke and hemorrhage. Circ: Arrhythmia and Electrophysiol. (2019) 12:e006841. 10.1161/CIRCEP.118.006841 30939908

[40] Landmesser U Schmidt B Nielsen-Kudsk JE Lam SCC Park JW Tarantini G Left atrial appendage occlusion with the AMPLATZER amulet device: periprocedural and early clinical/echocardiographic data from a global prospective observational study. EuroIntervention. (2017) 13:867–76. 10.4244/EIJ-D-17-00493 28649053

[41] Reddy VY Doshi SK Kar S Gibson DN Price MJ Huber K 5-year outcomes after left atrial appendage closure: from the PREVAIL and PROTECT AF trials. J Am Coll Cardiol. (2017) 70:2964–75. 10.1016/j.jacc.2017.10.021 29103847

[42] Kany S Bordignon S Piorkowski M Chen S Chun KRJ Tohoku S Retrieval of embolised left atrial appendage closure devices. EuroIntervention. (2021) 17:e178–80. 10.4244/EIJ-D-19-01088 32310128 PMC9724838

